# Outcomes After Repair of Pulmonary Atresia With Ventricular Septal Defect and Major Aortopulmonary Collateral Arteries: A Tailored Approach in a Developing Setting

**DOI:** 10.3389/fcvm.2021.665038

**Published:** 2021-04-14

**Authors:** Ming-Hui Zou, Li Ma, Yan-Qing Cui, Huai-Zhen Wang, Wen-Lei Li, Jia Li, Xin-Xin Chen

**Affiliations:** ^1^Department of Cardiovascular Surgery, Guangzhou Women and Children’s Medical Center, Guangzhou Medical University, Guangdong, China; ^2^Guangdong Provincial Key Laboratory of Research in Structural Birth Defect Disease, Department of Pediatric Surgery, Guangzhou Women and Children’s Medical Center, Guangzhou Medical University, Guangdong, China; ^3^Clinical Physiology Laboratory, Guangzhou Women and Children’s Medical Center, Institute of Pediatrics, Guangzhou Medical University, Guangdong, China

**Keywords:** pulmonary atresia, ventricular septal defect, major aortopulmonary collateral arteries, unifocalization, rehabilitation

## Abstract

**Objectives:** Pulmonary atresia with ventricular septal defect and major aortopulmonary collateral arteries (PA/VSD/MAPCAs) is complex and diverse that has led to a variety of treatment strategies. Experience has been largely obtained in the advanced countries. The clinical diversity is greater in China. We evaluated our surgical approaches and outcomes of these patients.

**Methods:** We reviewed 127 patients undergoing varied surgeries in our center in 2010–2019.

**Results:** Thirty patients underwent single-stage complete repair by unifocalizing MAPCAs and VSD closure (aged 3.9–131.4 months, median 22) with 3 (10%) early deaths. Ninety-seven underwent the first-stage rehabilitation strategy including systemic-to-pulmonary shunt in 29 (aged 0.5–144 month, median 8), and palliative RV-PA conduit in 68 (aged 2.2–209.6 months, median 14) with 5 (5.2%) early deaths. Eight-one patients (63.8%) eventually achieved complete repair with a median right/left ventricular (RV/LV) pressure ratio of 0.7 (ranged 0.4–1.0). Fourteen patients (11.0%) accepted palliation as final destination. Survival for the entire cohort was 89.5, 85.2, and 76.1% at 1, 5, and 10 years, respectively. Survival for those undergoing complete repair was 88.2 and 76.6% at 1 and 5 year, respectively. RV/LV pressure ratio ≥0.8 was risk factor for mortality (HR10.3, *p* = 0.003).

**Conclusions:** Our cohort, the largest from China, had distinctive clinical features with substantially wider age range and higher RV/LV pressure ratio. Using the combined approaches tailored to individual patients, complete repair was achieved in 64% of patients. The early and intermediate outcomes are acceptable compared to many of the previous reports.

## Introduction

Surgical strategies for pulmonary atresia with ventricular septal defects and major aortopulmonary collateral arteries (PA/VSD/MAPCAs) remain controversial. While some reported remarkable outcomes using the early complete unifocalization strategy, others recommended the rehabilitation strategy to promote development of native pulmonary arteries (1, 2). Currently both are used in many centers with favorable results (3–5).

To date, most reports are from the developed countries showing that early surgical intervention is ideal in infants and young children (1, 2, 6–8). However, in China and other developing countries, there is substantial heterogeneity in the age of patients at the first visit and consequent questionable potential for hypoplastic pulmonary arteries to develop and morphologic and structural variabilities in MAPCAs. Selection of surgical strategies for such patients continues to pose challenges. Therefore, we evaluated our strategy combining both rehabilitation and unifocalization and early and intermediate outcomes of the largest single-centered cohort of these patients in China.

## Patients and Methods

Following the approval by the Institutional Ethics Board, we retrospectively studied 127 consecutive patients from January 2010 to August 2019. All patients were diagnosed preoperatively using echocardiography and computed tomography. Patients who were initially judged suitable for complete repair underwent further cardiac catheterization and angiography to determine the anatomical features of the native pulmonary arteries, MAPCAs, and bronchopulmonary segment perfusion.

### Surgical Strategies

#### Initial Surgical Strategies

Based on the individualized characteristics of each patient, one of the following initial surgical strategies was chosen:

**1. Single-stage complete repair:** This involved right ventricular outflow tract (RVOT) reconstruction, complete unifocalization of MAPCAs, and VSD closure *via* median sternotomy or thoracotomy. RVOT reconstruction was performed with non-valve pericardial conduit, or valved polytetrafluoroethylene conduit, or bovine jugular valved conduit with the outer wall covered with a polyester film to prevent dilation (Balance Medical Technology Co Ltd., Beijing, China) since they became available in 2017. The decision making for complete repair requirements were: (1) preoperative hemoglobin saturation (SaO_2_) ≥85% at rest; (2) predicted total neopulmonary artery index (TNPAI) ≥150 mm^2^/m^2^ after unifocalization (9); (3) More than 3/4 of bronchopulmonary segments perfused by central pulmonary arteries after unifocalization since 2016 (≥2/3 before 2016); (4) pulmonary arterial flow study showing perfusion flow ≥3 L/min/m^2^ and mean pulmonary arterial pressure (mPAP) ≤25 mmHg since 2016 (1).

**2. Rehabilitation surgery:** For those with hypoplastic native pulmonary artery, we preferred to choose rehabilitation strategy. According to the diameter of intrapericardial native pulmonary artery, one of the followings was chosen:

**Systemic-to-pulmonary (S-P) shunt:** A central shunt was used for patients with poor native pulmonary artery development within the pericardium and a diameter ≤2.5 mm. Based on patient’s age and weight, a 4–5-mm polytetrafluoroethylene artificial blood vessel (Gore-Tex, W. L. Gore & Associates, Inc., Flagstaff, AZ, USA) was selected to connect the ascending aorta with the main pulmonary artery or the left and right pulmonary arteries. Since 2017, we used the Melbourne shunt in patients with severely hypoplastic pulmonary artery. A modified Blalock-Taussig shunt was performed in patients from other hospitals.

**Right ventricle to pulmonary artery (RV-PA) conduit connection:** Palliative RV-PA conduit connection was performed in patients with a native pulmonary artery diameter ≥3 mm. The conduits included non-valved autologous pericardial conduit and polytetrafluoroethylene (Gore-Tex) artificial blood vessel which were used in small infants (<10 kg weight) or older children with small native pulmonary arteries, and bovine jugular (Beijing Bairen Medical Technology Co Ltd., Beijing, China) and polytetrafluoroethylene valved conduit which was used in older children. The diameter of conduit was estimated according to this formula: Diameter (mm) = 0.325 × Weight (kg) + 4.629 (10).

#### Second-Stage and Multi-Stage Surgical Strategies

For patients who had received a S-P shunt, if pulmonary arterial development did not meet the criteria for complete repair as described above, conversion to RV-PA conduit connection was performed at 6 months after the shunt operation in order to further promote the pulmonary artery development.

For patients who had received a S-P shunt or RV-PA conduit, if pulmonary arterial development was satisfactory, complete repair was considered. Starting in 2016, we performed the intraoperative flow study as a functional measure of pulmonary vascular performance and candidacy for complete repair (1), with the additional borderline condition, i.e., mPAP at 25–30 mmHg, the VSD was closed with fenestration. But if mPAP > 30 mmHg, the VSD was left open. At the end of CPB, the RV/LV pressure ratio was measured. If the RV/LV pressure ratio >0.8, a fenestration was carried out. In patients who had undergone fenestration following the flow study, the RV/LV pressure ratio was found <0.8 with a left-to-right shunt at VSD by transesophageal echocardiography, closure of fenestration was performed. The VSD fenestration area was about 0.8–1.0 cm^2^/m^2^. Subsequent VSD closure was completed at the third-stage surgery or by transcatheter occlusion.

#### MAPCAs Management Strategy

For patients with preoperative angiography suggesting that MAPCAs had sufficient communication with native pulmonary arteries, MAPCAs were embolized by transcatheter occlusion or surgical ligation. For patients with MAPCAs as the only source of blood supply to the bronchopulmonary segment, MAPCAs were recruited to the native pulmonary arteries or the newly constructed pulmonary artery. In extreme cases, MAPCAs originated from the descending aorta at the T7–8 level were also connected using artificial blood vessels. Stenosis of MAPCAs was augmented with an autologous pericardial patch, or the proximal segment of the MAPCA up to the stenosis was excised when the distal segment has enough length that it can be recruited to other MAPCAs or the pulmonary artery.

### Follow-Up and Reintervention

Echocardiography and cardiac catheterization were performed every 3–6 months after each stage of surgery. Pulmonary arterial development, right and left ventricular function, right ventricular dilation, pulmonary regurgitation, and hemodynamic parameters were qualitatively described. The final clinical status including symptom, exercise tolerance, weigh gain, and death, was collected from the last available follow-up, as described by Amark et al. (11).

For patients who underwent palliative surgery, pulmonary arterial development, and MAPCA changes were assessed, and complete repair was performed if indicated as described above. Balloon dilation was performed for patients with stenosis of the anastomosis, left and right pulmonary arteries following palliative, or complete repair. For those with VSD fenestration, if SaO_2_ > 95%, and left to right shunt at VSD from echocardiography, and RV/LV pressure ratio <0.75 and Q_p_:Q_s_ > 1.5, the VSD fenestration was occluded using transcatheter device.

### Statistical Analysis

Data were expressed as mean ± SD, median (range), or frequency (%) when appropriate. Between-group differences were compared using a *t*-test or chi-squared test when appropriate. The Kaplan-Meier method was used to estimate survival time, and the log-rank test was used to compare differences in survival rates. Univariate Cox regression analysis was performed, and variables with a *P*-value <0.1 were included. A forward stepwise regression method was used based on maximum likelihood estimation. A *P*-value <0.05 was considered significant. SPSS 22.0 (IBM, Armonk, NY) was used for statistical analyses.

## Results

### Patient Characteristics

In the 127 patients, the preoperative SaO_2_ was 77% (range: 45–94%). The median number of MAPCAs per patient was 3.9 (range: 2–7). There were 116 patients with native pulmonary artery confluence, three with intrapericardial pulmonary artery non-confluence, and eight with complete absence of native pulmonary arteries (Table 1).

**Table 1:** Patient characteristics at the initial admission (*n* = 127).

**Variable**	**Value**
Admission age (months)	16 (0.5–209.6)
Weight (kg)	8.5 (2.6–53.1)
Gender (male/female)	75/52
Premature (*N*, %)	14 (11.0%)
Non-cardiac anomaly (N, %)	46 (36.2%)
Morphologic characteristics (*N*, %)	
Aberrant subclavian artery	16 (12.6%)
Coronary artery abnormality	9 (7.1%)
Aortic valve regurgitation	16 (12.6%)
Trachea/bronchial abnormality	17(13.4%)
Pulmonary arterial anatomy	
Absent main pulmonary artery	90 (70.9%)
Non-confluent pulmonary arteries	3 (2.4%)
Absent native pulmonary arteries	8 (6.3%)
LPA main branch stenosis^*^	14 (11.0%)
RPA main branch stenosis^*^	6 (4.7%)
MAPCAs anatomy	
Total number	494
Number per patient	3.9 (2–7)
Origin (*N*, %)	
Subclavian artery	39 (7.9%)
Descending aorta	445 (90.1%)
Aorta arch	7 (1.4%)
Coronary artery	3 (0.6%)
Bifurcation (*N*, %)	59 (11.9%)
Number of segments supplied	6.5 (3–12)

* *defined as the pressure gradient >30 mmHg*.

### Surgical Algorithm and Early Outcomes

Thirty patients underwent single-stage complete repair at a median age of 22 months (range: 3.9–131.4 months), which was significantly younger than that of multi-stage group (*P* < 0.0001). Of these, eight patients had absent native pulmonary arteries. The median Nakata index of 22 patients with hypoplastic native pulmonary artery was 153.3 mm^2^/m^2^ (range: 24.7–279.5 mm^2^/m^2^). A median of 2.9 (range: 1–5) MAPCAs per patient in this group were recruited, and 0.7 (range: 0–4) MAPCAs were ligated intraoperative because of dual supply. Materials for right ventricular outflow tract reconstruction included bovine jugular valved conduit in 19, non-valved autologous pericardial conduit in eight, and polytetrafluoroethylene (Gore-Tex) valved conduit in three patients. Twenty-one patients underwent VSD complete closure, and nine with VSD patch fenestration. Early postoperative death occurred in three patients (10.0%) (the RV/LV pressure ratio >0.9). There was no statistical difference in the early mortality between single-stage and multi-stage complete repair group (10 vs. 5.9%, *P* = 0.665). The anatomical characteristics and operative details of patients underwent complete repair are summarized in Table 2.

**Table 2:** Characteristics and operative details of patients undergoing complete repair.

**Variable**	**Multi-stage complete repair** **(*n* = 51)**	**Single-stage complete repair** **(*n* = 30)**	***P*-value**
Age at complete repair (months)	47 (11.1–222.3)	22 (3.9–131.4)	<0.0001
SaO_2_ (%)	83 (75–89)	85 (78–94)	0.715
Total numbers of MAPCAs	194	126	
Numbers of MAPCAs per patient	3.8 (2–7)	4.2 (2–6)	0.885
Segments supplied by MAPCAs	6.0 (3–10)	7.0 (4–12)	0.629
Nakata index of native PA before first palliative surgery (mm^2/^m^2^)	44.5 (8.2–84.9)	–	–
Nakata index of native PA at complete repair (mm^2/^m^2^)	192.6 (110.4–306.6)	153.3 (24.7–279.5)	0.067
Intraoperative details			
Unifocalization of MAPCAs			
Number of recruited per patient	2.8 (0–5)	3.4 (1–5)	0.385
Number of ligated per patient	1.5 (0–5)	0.7 (0–4)	0.091
Median sternotomy	43 (84.3)	28 (93.3)	0.07
Thoracotomy	8 (15.7)	2 (6.7)	0.07
RVOT reconstruction (*N*, %)			
Bovine jugular valved conduit	24 (47.6%)	19 (63.3%)	0.129
Valved PTFE graft	5 (9.8%)	3 (10.0%)	
Non-valved autograft pericardial conduit	14 (27.5%)	8 (26.7%)	
Bovine pericardial patch	8 (15.7%)	0	
Closure of VSD/ASD (*N*, %)			
VSD complete closure	38 (74.5%)	21 (70%)	0.797
VSD fenestration	13 (25.5%)	9 (30%)	0.797
ASD complete closure	10 (19.6%)	4 (3.3%)	0.554
ASD fenestration	41 (80.4%)	26 (86.7%)	0.554
Concomitant procedure (*N*, %)			
Aortic valvuloplasty	2 (3.9%)	1 (3.3%)	0.999
Aortic valve replacement	1 (2.0%)	0	0.999
Mitral valvuloplasty	6 (11.8%)	2 (6.6%)	0.704
Glenn takedown	4 (7.8%)	0	0.291
Occluder/coil takeout	3 (5.9%)	0	0.292
RV/LV pressure ratio after repair	0.72 ± 0.14	0.67 ± 0.17	0.202
Cardiopulmonary bypass time (min)	226 ± 94	249 ± 80	0.268
Aortic cross-clamp time (min)	83 ± 29	63 ± 21	0.001
Mechanical ventilation time (hour)	82 ± 106	79 ± 105	0.897
ICU stay (day)	9 ± 10	10 ± 9	0.743
Hospital stay after surgery (day)	21 ± 14	24 ± 18	0.369
Early mortality (*N*, %)	3 (5.9%)	3 (10%)	0.665

Ninety-seven patients underwent the first-stage palliative surgery to rehabilitate native pulmonary arteries. Of these, 29 patients underwent S-P shunt with a median Nakata index of 33.5 mm^2^/m^2^ (range: 11.2–60.6 mm^2^/m^2^); 13 of them received a modified Blalock-Taussig shunt and 16 received a central shunt (including five Melbourne shunts) at a median age of 8 months (range: 0.5–144 months). The remaining 68 patients underwent palliative RV-PA conduit connection at a median age of 14 months (range: 2.2–209.6 months) and the median Nakata index was 45.2 mm^2^/m^2^ (range: 8.2–184.9 mm^2^/m^2^). Conduit materials included non-valved autologous pericardial conduits in 60 patients, Gore-Tex artificial blood vessels in six, and bovine jugular valved conduit in two. Five patients (5.2%) died within 30 days after the first-stage palliative surgery, including three with S-P shunts (10.3%) ((1) center shunt and (2) the Melbourne shunt) and two with RV-PA conduit connection (2.9%). The group with RV-PA connection trended lower early mortality than that of S-P shunt (p = 0.063).

At a median of 24 months (range: 6–116 months) after the first-stage S-P shunt, 13 patients achieved complete repair (three had VSD fenestration) and two were awaiting next-stage surgery. Owing to unsatisfactory native pulmonary artery development, 11 patients were converted to RV-PA conduit connection with two simultaneous unifocalization of MAPCAs. Among these, four underwent complete repair 1–3 years after the conversion and five awaiting the complete closure of VSD; two died early after surgery of cardiac dysfunction as a result of left anterior descending coronary artery injury and poor pulmonary arterial development, respectively. In total, 58% (17/29) achieved complete repair after the first-stage palliative surgery.

**Figure 1:**
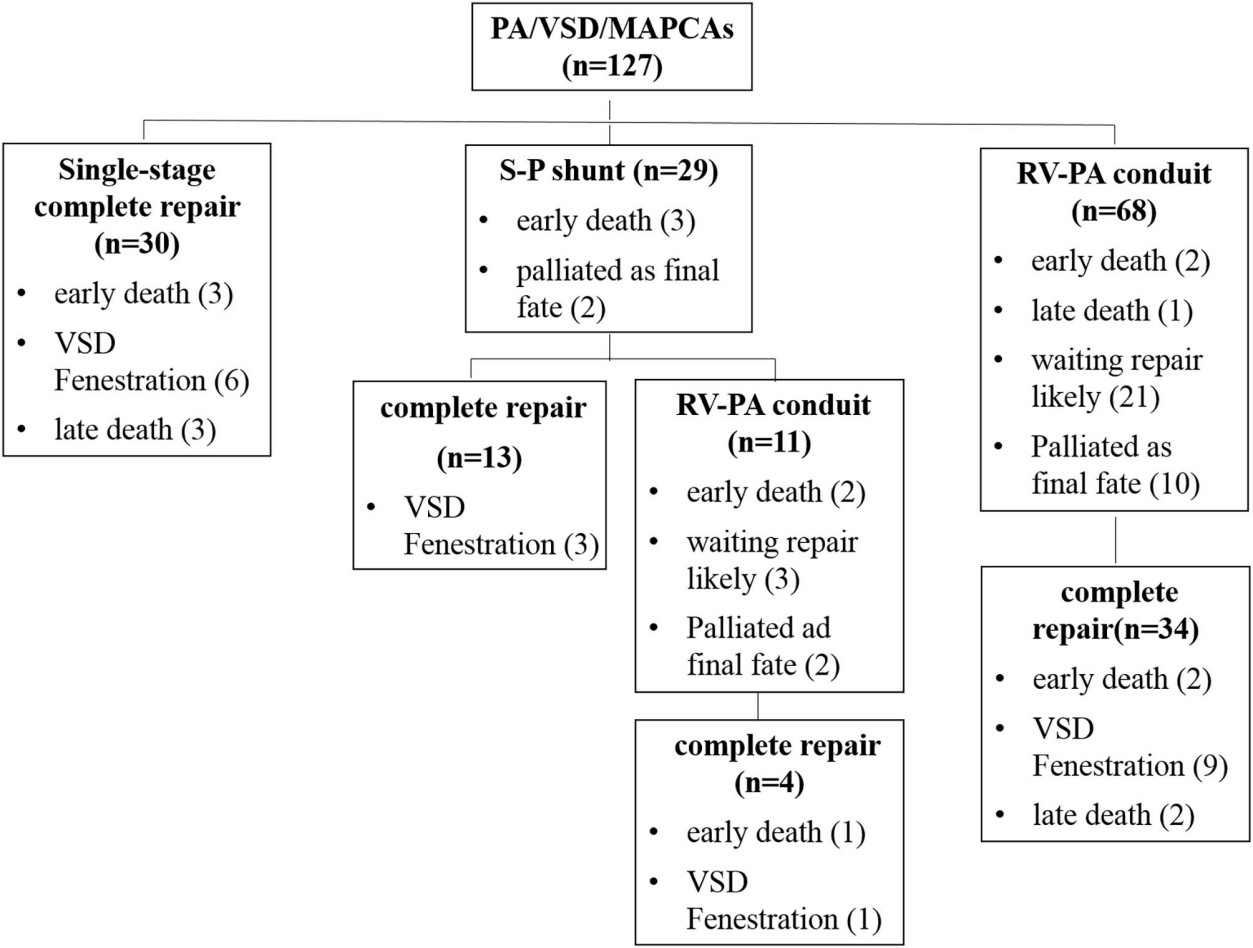
The outcomes of 127 PA/VSD/MAPCAs patients with combined strategy.

In patients undergoing the palliative RV-PA conduit connection, 34 (50%) underwent complete repair after 18 months (range: 6–100 months) at a median age of 47 months (range: 22–222 months) with 3 (8.8%) early deaths. In the 34 patients, the native pulmonary artery Nakata index increased from the median 55.6 mm^2^/m^2^ (range: 18.2–84.9 mm^2^/m^2^) at the first palliative surgery to the median 126.6 mm^2^/m^2^ (range:102.2–176.3 mm^2^/m^2^) at complete repair. There was one late death at 5 months postoperatively due to purulent meningitis. In the remaining unrepaired patients, 21 (30.9%) had the subsequent complete repair anticipation, and 10 (14.7%) were felt to never be suitable for close VSD due to poor distal pulmonary artery development (Figure 1).

### Follow-Up, Reintervention, and Intermediate Outcomes

At the latest follow-up, eighty-one patients (63.8%) underwent complete repair with 6 (7.4%) early deaths (3 of 30 single-stage and 3 of 51 multi-stage complete repair) (Figure 1). The surviving 75 patients included 16 with VSD fenestration. The RV/LV pressure ratio was 0.67 ± 0.17 for single-stage complete repair and 0.72 ± 0.14 for multi-stage complete repair (*P* = 0.2). There were five late deaths; three of them were after single-stage complete repair, one developed RV-PA conduit stenosis 5 years after complete repair and died after conduit replacement due to intracranial infection; two had sudden death (RV/LV pressure ratio ≥0.9). The remaining two deaths were due to right ventricular failure after multi-stage complete repair.

Reintervention was performed in 16 patients, including cardiac catheterization in 12 patients and surgery in four. Balloon dilation was performed in five patients with right and/or left pulmonary artery stenosis in 6–10 months after palliative RV-PA conduit connection, and four with stenosis of unifocalized MAPCAs in 1–3 years after complete repair. Transcatheter VSD occlusion was performed in three patients in 1 month−1 year after complete repair. Three patients with RVOT obstruction underwent surgical replacement; one with severe reflux of the right pulmonary artery conduit underwent subsequent valved conduit replacement. There was no death after reintervention.

**Figure 2:**
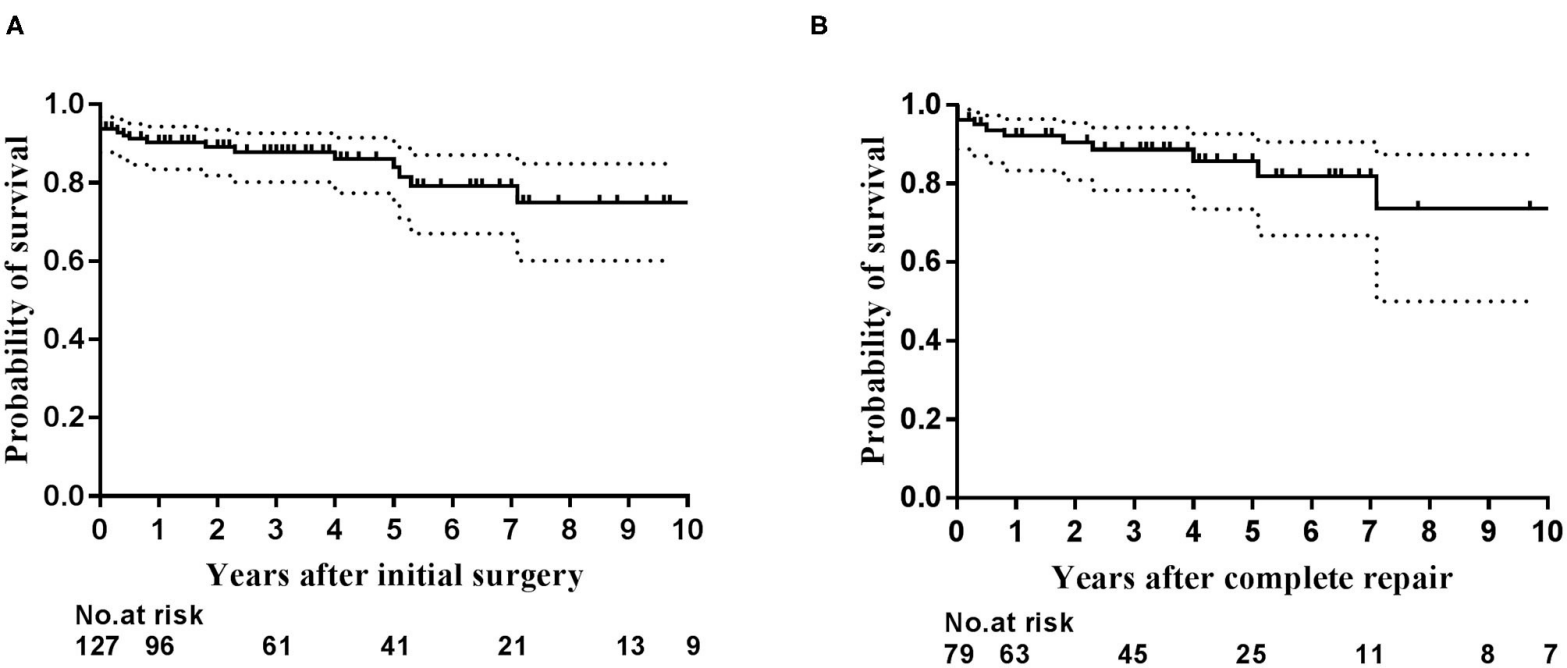
The Kaplan–Meier curves depict estimated survival. **(A)** Survival for the entire cohort of 127 patients after initial operation; **(B)** Survival for patients after complete repair.

The overall 1-, 5-, and 10-year survival rates after initial operation were 89.5% (95% CI: 84.0–95.0%), 85.2% (95% CI: 78.1–92.3%), and 76.1% (95% CI: 64.1–88.1%), respectively (Figure 2A). The 1- and 5-year survival rates after complete repair were 88.2% (95% CI: 81.0–95.5%), and 76.6% (95% CI: 0.4–96.8%), respectively (Figure 2B). Univariate regression analysis showed postoperative RV/LV pressure ≥0.8 (HR: 10.35, 95% CI: 2.19–48.93, *P* = 0.003), single-stage complete repair (HR: 3.49, 95% CI: 1.06–11.49, *P* = 0.039) and the era of complete repair surgery before 2016 (HR: 4.48, 95% CI: 1.25–16.03. *P* = 0.021) were risk factors for mortality after repair. Of note, the three early postoperative deaths following single-stage complete repair occurred before 2016. Multivariate regression analysis showed postoperative RV/LV pressure ≥0.8 (HR: 10.35, 95% CI: 2.19–48.93. *P* = 0.003) was only risk factor for death. The early mortality rate after 2016 was significantly lower than that of the 2010–2015 period (35.0 vs. 6.7%, *P* < 0.0001). Despite the high risk of re-stenosis of pulmonary arteries and branches, most patients showed good clinical status with no or mild exercise limitation (Table 3). The clinical status between single- and multi-stage complete repair groups was not significantly different (*P* = 0.24). Due to the poor pulmonary artery vasculature, 14 (11.0%) (10 in RV-PA conduit connection, 4 in S-P shunt) cases were felt to never be suitable for VSD closure and the palliation was likely to be their final destination.

**Table 3:** Patient characteristics at follow-up after complete repair.

**Variable**	**Multi-stage complete repair** **(*n* = 48)**	**Single-stage complete repair** **(*n* = 27)**	***P*-value**
Follow-up time (year)	2.0 (0.6–8.9)	1.5 (0.5–6.5)	0.028
SaO_2_ (%)	94 (90–96)	96 (93–97)	0.181
Pulmonary arterial anatomy (N, %)			
Main PA stenosis^*^	2 (4.2%)	2 (7.4%)	0.623
LPA main branch stenosis	11 (22.9%)	2 (7.4%)	0.133
RPA main branch stenosis	14 (29.2%)	2 (7.4%)	0.038
Bilateral PA stenosis	5 (10.4%)	1 (3.7%)	0.403
Recruited MAPCAs stenosis^*^	10 (20.8%)	6 (22.2%)	1.0
Intracardiac anatomy (N, %)			
Residual VSD fenestration	9 (18.8%)	3 (11.1%)	0.516
Pulmonary regurgitation	17 (35.4%)	4 (14.8%)	0.061
RV dilation	28 (58.3%)	6 (22.2%)	0.002
Significant Reduced RV function	1 (2.1%)	0	1.0
Hemodynamic measurements			
PA systolic pressure (mm Hg)	52 ± 12	49 ± 10	0.639
RV/LV pressure ratio	0.58 ± 0.12	0.55 ± 0.11	0.336
Reoperation (N, %)	2 (4.2%)	2 (7.4%)	0.623
Reintervention (N, %)	4 (8.3%)	1 (3.7%)	0.645
Clinical status			
Late death (%)	2 (4.2%)	3 (11.1%)	0.244
Asymptomatic without limitations	28 (58.3%)	19 (70.4%)	
Mild exercise intolerance	17 (35.4%)	5 (18.5%)	
Severe exercise intolerance	1 (2.1%)	0 (0)	
Poor weight gain	12 (25.0%)	9 (33.3%)	0.595

* *defined as the pressure gradient more than 30 mmHg*.

## Discussion

This study reviewed our experience that combined the distinctive patient characteristics in China with literature reports from advanced countries in the management of 127 patients with PA/VSD/MAPCAs in our center over the past decade, representing the developing course of surgical, and interventional management of these patients. This is the largest cohort report from China and the third largest in the world to date. Our data showed that despite of even greater clinical diversity, particularly older age, and higher RV/LV pressure ratio after complete repair, 63.8% of patients ultimately achieved complete repair with satisfactory early and intermediate outcomes.

Regarding the shunt, our strategy was to use a S-P shunt for those with native right and left pulmonary artery diameters of ≤2.5 mm. If, at 6 months after surgery, pulmonary arterial development was unsatisfactory, we converted to RV-PA conduit connection. In this group, 44.8% (13/29) achieved complete repair at the second stage, while 37.9% (11/29) showed unsatisfactory pulmonary arterial development and were converted to RV-PA conduit connection at the second stage. Four of these patients achieved complete repair at the third stage, making the cumulative complete repair rate 58.6% (17/29).

The palliative RV-PA connection includes the use of various conduits to reconstruct RV-PA continuity. We preferred non-valved autologous pericardial conduit in small infants to reconstruct RV-PA connection. Its advantages include easily suture, less bleeding, no extra cost, and longer term of patency. When the autologous pericardium is not available, the Gore-tex artificial blood vessel is considered. Hovever, for older children with hypoplastic pulmonary arteries, we preferred using a valved conduit, such as, bovine jugular valved conduit with the outer wall covered with a polyester film to prevent dilation or polytetrafluoroethylene (Gore-Tex) valved conduit, because high distal resistance may cause significant regurgitation and lead to right ventricular dysfunction. Besides, in order to avoid overflow of the pulmonary circulation, the diameter of conduit is generally restrictive calculated from the established formula (10). Of the 68 patients undergoing palliative RV-PA conduit connection, we found that the risk of unexpected over-circulation and significant regurgitation and right ventricular dysfunction is low. Despite of the older age at the first-stage palliative surgery (age range 2.2–209.6 months (median 14 months) in our patients as compared to the age range 2–6 months in previous reports) (12–14), RV-PA conduit connection still promoted native pulmonary arterial development as indicated by the development in Nakata index, and 47.1% (32/68) patients achieved complete repair.

A valved conduit is adopted at the complete repair in the current era. Nonetheless, if the development of pulmonary vascular bed is good without elevated pulmonary artery pressure and pulmonary vascular resistance, i.e., the RV / LV pressure ratio <0.8 at the end of CPB and stable systemic arterial blood pressure, a non-valved conduit was applicable, particularly when the valved conduit was unavailable as in our experience. This situation is similar to the use of a transannular patch in the complete repair of tetralogy of Fallot. In our cohort, 8 (26.7%) in one-stage and 22 (43.2%) in multistage complete repair used non-valved conduit, and in the intermediate follow-up, only 1 (2.1%) of patients in multistage complete repair group showed significant right ventricular dilation and dysfunction.

Regarding the treatment strategies for MAPCAs, early complete unifocalization, even in patients with absence of the native pulmonary arteries, was recommended to obtain low-pressure pulmonary circulation (15, 16). Patients whose MAPCAs had sufficient communication with native pulmonary arteries underwent transcatheter occlusion or surgical ligation. Recruitment was performed wherever possible for MAPCAs that supply individual bronchopulmonary segments during complete repair. Some centers such as Stanford and Birmingham prefer the S-P shunt or RV-PA conduit connection at early complete unifocalization and have reported excellent results (4, 16). However, our earlier experiences before 2010 (before the study period) with this strategy showed a fairly high early mortality. Subsequently we adopted the rehabilitation strategy.

The indications for complete repair remain controversial. In our experience, among the four requirements described above, two are more important, i.e., more than 3/4 of bronchopulmonary segments perfused by central pulmonary arteries after unifocalization, and the result of the flow study. SaO_2_ and Nakata index, on the other hand, are less so. In total, there were three early deaths and three late deaths after single-stage complete repair, all with the RV/LV pressure ratio ≥0.8. Of note, the three early deaths following single-stage complete repair occurred before 2016. The statistical result showing single-stage complete repair as a risk factor is not applicable to later era.

In the entire cohort, the 1-, 5-, and 10-year survival rates were 89.5, 85.2, and 76.1%, respectively. The 1- and 5-year survival rates after complete repair were 88.2 and 76.6%, respectively. The results are not optimal, nonetheless acceptable when compared to many of the previous reports (1, 2, 8, 16–18). As described above, some progressions were made since 2016, including the implementation of the pulmonary arterial flow study to predict VSD closure and more strict criteria for complete VSD closure in 2016, the availability of valved conduit in 2017, and implementation of follow-up cardiovascular angiography and balloon dilation in 2019. As shown in our data, mortality rate significantly reduced from 35.0% in 2010–2015 to 6.7% since 2016.

### Limitations

Our study has several limitations. (1) This is a single-centered study. Our center has the most cases of PA/VSD/MAPCAs in China, thus, our results do not represent the overall practice and outcomes in China as there is large disparity of patient populations and treatment strategies among centers. (2) The 10-year study period represents our learning course whereby the surgical strategies were continuously evolved. (3) The follow-up and catheterization protocol that is still being refined.

## Conclusion

Using an approach tailored to the distinctive patient characteristics in China, about two-thirds of PA/VSD/MAPCAs patients achieved complete repair with satisfactory early and intermediate survival rates that, although suboptimal, are acceptable compared to many of the previous results, despite of older age, and higher RV/LV pressure ratio after complete repair. Refinements and improvements in perioperative and follow-up management are warranted to further improve clinical outcomes of this particularly complex group of patients.

## Data Availability Statement

The original contributions presented in the study are included in the article/supplementary material, further, inquiries can be directed to the corresponding author/s.

## Ethics Statement

The studies involving human participants were reviewed and approved by Guangzhou Women and Children’s Medical Center. Written informed consent to participate in this study was provided by the participants’ legal guardian/next of kin. Written informed consent was obtained from the individual(s), and minor(s)’ legal guardian/next of kin, for the publication of any potentially identifiable images or data included in this article.

## Author Contributions

M-HZ: conceptualization, data curation, investigation, and writing - review & editing. LM: conceptualization, data curation, and writing - review & editing. Y-QC: data curation, formal analysis, and investigation. H-ZW: data curation and statistical analysis. W-LL: writing - original draft. JL: conceptualization and writing - review & editing. X-XC: conceptualization, investigation, and supervision. All authors contributed to the article and approved the submitted version.

## Conflict of Interest

The authors declare that the research was conducted in the absence of any commercial or financial relationships that could be construed as a potential conflict of interest.

## Abbreviations

ASD: Atrial septal defect
LV: Left ventricle
PA/VSD/MAPCAs: ulmonary atresia with ventricular septal defects and major aortopulmonary collateral arteries
RV: Right ventricle
RV-PA: Right ventricle to pulmonary artery
RVOT: Right ventricular outflow tract
TNPAI: Total neopulmonary artery index.
